# Cancer mortality and morbidity in employees of the United Kingdom Atomic Energy Authority, 1946-86.

**DOI:** 10.1038/bjc.1993.113

**Published:** 1993-03

**Authors:** P. Fraser, L. Carpenter, N. Maconochie, C. Higgins, M. Booth, V. Beral

**Affiliations:** Department of Epidemiology and Population Sciences, London School of Hygiene & Tropical Medicine, UK.

## Abstract

In further analyses of a cohort of 39,718 United Kingdom Atomic Energy Authority employees after 7 more years follow-up, cancer mortality, based on 1,506 deaths in 1946-86, was 20% below the national average. Prostatic cancer mortality showed a statistically significant association with external radiation exposure, largely confined to men who were also monitored for internal contamination by radionuclides other than plutonium. Prostatic cancer mortality was highest in radiation workers at Winfrith. In women monitored for radiation exposure, mortality from cancer of the uterus (including the cervix uteri) was increased relative to other employees, and, showed a statistically significant association with external radiation exposure. While there were some other statistically significant results, as would be expected by chance alone when multiple comparisons are made, there were no other cancer sites with consistently exceptional findings. Point estimates for risk associated with increasing exposure to radiation suggest a decrease of four deaths per 10(4) person-years per Sv for leukaemia and an increase of 20 deaths for all cancers except leukaemia, but confidence intervals indicate that a wide range of values are compatible with the data. Cancer morbidity based on 1,699 registrations in 1971-84 was 12% below the national average. Findings from site-specific analyses largely replicated those of the mortality analyses.


					
Br. J. Cancer (1993), 67, 615-624                                                                 ?  Macmillan Press Ltd., 1993

Cancer mortality and morbidity in employees of the United Kingdom
Atomic Energy Authority, 1946-86

P. Fraser', L. Carpenter', N. Maconochie', C. Higgins', M. Booth' & V. Beral2

'Epidemiological Monitoring Unit, Department of Epidemiology and Population Sciences, London School of Hygiene & Tropical
Medicine, Keppel Street, London WCIE 7HT; 2lmperial Cancer Research Fund Cancer Epidemiology Unit, Gibson Building,
Radcliffe Infirmary, Oxford OX2 6HE, UK.

Summary In further analyses of a cohort of 39,718 United Kingdom Atomic Energy Authority employees
after 7 more years follow-up, cancer mortality, based on 1,506 deaths in 1946-86, was 20% below the national
average. Prostatic cancer mortality showed a statistically significant association with external radiation
exposure, largely confined to men who were also monitored for internal contamination by radionuclides other
than plutonium. Prostatic cancer mortality was highest in radiation workers at Winfrith. In women monitored
for radiation exposure, mortality from cancer of the uterus (including the cervix uteri) was increased relative to
other employees, and, showed a statistically significant association with external radiation exposure. While
there were some other statistically significant results, as would be expected by chance alone when multiple
comparisons are made, there were no other cancer sites with consistently exceptional findings. Point estimates

for risk associated with increasing exposure to radiation suggest a decrease of four deaths per 104 person-years

per Sv for leukaemia and an increase of 20 deaths for all cancers except leukaemia, but confidence intervals
indicate that a wide range of values are compatible with the data.

Cancer morbidity based on 1,699 registrations in 1971-84 was 12% below the national average. Findings
from site-specific analyses largely replicated those of the mortality analyses.

Follow-up data for a further 7 years have accrued since we
first described the mortality of employees of the United
Kingdom Atomic Energy Authority (UKAEA), 1946-79
(Beral et al., 1985). Cancer mortality then was 21% below
the national average and prostatic cancer was the only malig-
nancy with clearly increased mortality in relation to radiation
exposure. However, the duration of follow-up to the end of
1979 was only 16 years on average, and small numbers of
deaths from some cancers yielded imprecise estimates which
were consistent with a wide range of effects. In order to
increase precision and allow for longer latency, we have
continued to collect follow-up data on the entire UKAEA
study population, including employees still in service on 1
January 1980. Analyses of mortality data from 1946 to 1986,
and cancer registration data from 1971 to 1984, are reported
here with special reference to prostatic cancer, other genital
tract cancers, and malignancies such as multiple myeloma
which have been associated with radiation exposure in other
studies of nuclear industry workers (Smith & Douglas, 1986;
Gilbert et al., 1989).

Methods

The design and methods of data collection and validation in
the UKAEA mortality study have been described previously
(Fraser et al., 1985) and a fully account of the methods used
in this report will be published elsewhere (Fraser et al., in
preparation).

Study population, personnel data andfollow-up

The study population comprised all employees of the UKAEA
establishments at Harwell, London, Culham, Dounreay and
Winfrith who were ever employed between 1 January 1946
and 31 December 1979. Details of all employees were submit-
ted for tracing to the National Health Service Central
Registers (NHSCRs) in Southport and Edinburgh. For sub-
jects recorded as having died, both the underlying and
associated causes of death as stated on the death certificate
were coded to the eighth (for deaths during 1946-78) and

ninth (for deaths during 1979-86) revisions of the Interna-
tional Classification of Diseases (ICD) by the Office of Popu-
lation Censuses and Surveys. Coded death certificates and
notifications of emigrations and cancers registered since 1971
were obtained. When a subject could not be traced at the
NHSCRs, identifying particulars were sent to the Depart-
ment of Social Security's (DSS) Records Branch in Newcastle
for ascertainment of vital status.

The UKAEA's records of deaths in service and among
members of their pension scheme provided a cross-check on
the completeness of notification of death and sometimes
information of assistance in tracing deaths at the NHSCRs.
Further checks on the completeness of notification of cancer
deaths and random samples of non-cancer deaths were car-
ried out at NHSCR in Southport. Deaths notified in the
UKAEA study population were also cross-checked against
deaths in radiation workers notified to the National Radio-
logical Protection Board (NRPB) (Kendall. et al., 1992a,b).
Finally, an extensive computerised check on the vital status
of UKAEA study members using National Insurance numbers
was carried out by the DSS' Information Technology Ser-
vices Agency.

Radiation data

Information on employees who had been monitored for
exposure to radiation during 1946-85 was obtained from
UKAEA records. Data abstracted for each employee includ-
ed a yearly summary of external whole body radiation expo-
sure (X-rays, gamma rays and neutrons) including exposures
accumulated in previous employments when these had been
notified to the UKAEA. Annual whole body exposures were
cumulated adjusting exposures in 1946-79 for below-thres-
hold measurements as described by Inskip et al. (1987). After
1979 no adjustments were made as this source of error
became negligible. Adjustments were made throughout the
study period for missing values due to lost or damaged films
by incorporating an appropriate fraction of the worker's
annual recorded exposure in that year. Information on inter-
nal exposure from radionuclides was generally limited to
noting the years in which subjects were monitored for pos-
sible internal contamination by tritium, plutonium, or other
unspecified radionuclides. Associated radiation exposures
were not included in measures of whole body exposure except
from tritium at Harwell from 1977.

Correspondence: P. Fraser.

Received 11 May 1992; and in revised form 19 October 1992.

Br. J. Cancer (1993), 67, 615-624

'?" Macmillan Press Ltd., 1993

616    P. FRASER et al.

Statistical analysis

Person-years at risk were calculated from each worker's first
day of employment by the UKAEA (or from 1 January 1946
for workers recruited before that date) to 31 December 1986
or the date of emigration, death, or the last date traced if
any of these preceded 1 January 1987. Person-years-at-risk
and deaths were stratified by sex, age in 15 groups, calendar
year both in single years and in nine groups, establishment in
three groups (Harwell with Culham and London, Dounreay
and Winfrith), and social class in six groups. Radiation
exposure was treated as a time-dependent variable in all
analyses. In order to allow for the delayed effects of radiation
exposure, the exposures were lagged in some analyses by 2
years for leukaemia and 10 years for other causes of death
using the same method as before (Beral et al., 1985). Some
analyses were repeated with a 5-year lag.

All mortality analyses were based on the underlying cause
of death. Deaths for which an underlying cause could not be
ascertained were included in analyses of deaths from all
causes but not in cause-specific analyses. Age, sex and single-
year specific death rates for England and Wales were used to
calculated expected deaths and standardised mortality ratios
(SMR). Exact 95% confidence intervals (CI) and statistical
significance levels for SMRs were obtained using the Poisson
distribution (Bailer & Ederer, 1964) unless the numbers of
observed deaths was 200 or more, when approximate signifi-
cance levels were derived from the standard chi-squared test
(Breslow & Day, 1987).

Rate ratios (RR) adjusted for age, sex, calendar period,
establishment and social class, and approximate 95% CIs,
were estimated by the method of maximum likelihood using
the GLIM computer package (Baker & Nelder, 1982). Statis-
tical significance of the RRs was tested using the likelihood
ratio statistic and checked using the score statistic (Breslow &
Day, 1987). When the total number of deaths contributing to
the RR was less than 20, exact confidence intervals and
significance levels were generated from the stratum-specific
deaths and person-years using the likelihood for binomial
data.

For workers with a radiation record, the relation between
level of external whole body radiation exposure and mortal-
ity was examined without reference to national rates. An
overall chi-squared statistic was obtained to test for a linear
trend across five levels of exposure stratified by age, sex,
calendar period, establishment and social class (Breslow &
Day, 1987). When the resultant test statistic was based on a
total number of deaths of 20 or less, probability values were
checked using 10,000 simulations as described elsewhere
(Beral et al., 1988). Changes in cancer risk associated with
increasing exposure to radiation were estimated using an
additive relative risk model. Excess relative risks and absolute
risks (and their 95% CIs) were estimated from this model
using maximum likelihood methods described by Gilbert
(1989).

The cancer registration data were analysed using methods
similar to those employed for mortality. Cancers first identi-
fied through a death certificate as the underlying or an
associated cause of death were included in all internal (within
workforce) comparisons, the date of death being used as a
surrogate for the registration date. Where an ill-defined,
secondary or unspecified neoplasm was registered but a pri-
mary malignant neoplasm was specified on the death certifi-
cate, the date of registration was retained but the primary
site was substituted in all internal analyses. For compar-
ability with national cancer registration rates in England and
Wales, cancers first identified through a death certificate in

1971-73 were not included in calculations of standardised
registration ratios (SRR).

P-values obtained from tests of statistical significance are
quoted as two-sided throughout. Separate analyses were per-
formed for many individual anatomical sites - 29 for cancer
mortality and 14 for cancer registrations. Some statistically
significance results may therefore be expected to occur on the
basis of chance alone. In interpreting the findings, those for

which similar results have not been described in other studies
are considered as being likely to be due to chance, except
when the P-value is less than 0.01. Where similar results have
been described before, attention is drawn to findings with a
P-value of less than 0.05 in the expected direction. Confi-
dence intervals for SMRs and RRs are quoted throughout
the text, to give an indication of the range of possible values
for the true effect, and to examine its consistency with that of
other studies.

Results

The total study population numbered 39,869. Of these, 151
lacked essential information such as sex, date of birth, or
dates of employment. These individuals could neither be
traced nor included in the analyses; only one had a radiation
record but no recorded dose. The analyses presented here are
therefore based on 39,718 subjects - 172 more than the
39,546 subjects analysed previously (Beral et al., 1985). Of
these, 29,085 (73%) were men and 21,545 (54%) had a
radiation record, of whom 19,760 (92%) were men. The
average length of follow-up was 22 years. Subjects with a
radiation record had been followed-up for an average of 23
years and those without for an average of 21 years.

The collective external radiation exposure in the UKAEA
study population from 1946-85 was 862 Sv giving an aver-
age exposure of 40 mSv per monitored worker. The distribu-
tion was highly skewed with half (10,806) of the 21,545
workers with a radiation record having a final cumulative
whole body exposure of 10 mSv or more, 10% (2,211) a final
exposure exceeding 100 mSv, and less than 1% (150) a final
exposure exceeding 500 mSv. Exposures varied between esta-
blishments, average exposures at Winfrith (56 mSv) and
Dounreay (49 mSv) being higher than at Harwell with Cul-
ham and London (32 mSv). Among workers who had a
radiation record, 7,480 (35%) were monitored for internal
contamination by radionuclides; 1,702 (8%) were monitored
for tritium, 3,564 (17%) for plutonium, and 6,412 (30%) for
other unspecified radionuclides. Fifty-one percent (3,834) of
radionuclide-monitored workers fell into more than one of
these categories.

A total of 5,509 deaths (14% of the study population) had
occurred by 31 December 1986. The cause of death had been
confirmed by necropsy in 781 subjects with radiation records
(25.9%) and 655 subjects without (26.3%). There were 1,506
deaths from cancer and 45 deaths for which the underlying
cause could not be ascertained. A total of 1,706 ex-employees
(4% of the study population) were reported to have emigrat-
ed by 31 December 1986. Of the 111 (0.3%) ex-employees
who were lost to follow-up after leaving the UKAEA, only
ten had a radiation record.

Among the 28,594 study members (72% of the study
population) whose records were identified through National
Insurance numbers, DSS reported belatedly 82 deaths in the
UK during 1970-86 which had not been notified by the
NHSCRs, 50 of them in monitored workers. Three more
deaths in Dounreay radiation workers came to light in the
cross-check against deaths notified to NRPB. The distribu-
tion of all 53 missed deaths in radiation workers across dose
categories was not dissimilar to the distribution of the 3,021
notified deaths in the study population so their omission
from the analyses is unlikely therefore to have resulted in
serious bias. An examination of deaths by year suggested a
deficit in the numbers of deaths notified by the NHSCRs in

1985, and possibly in 1986. The proportions of missed deaths
in 1985 and 1986, 3.5% and 2.9% respectively, whilst above
the annual average were not extreme.

Mortality compared with national rates: standardised mortality
ratios

The standardised mortality ratio was 78 (95% CI 76-80) for
all causes of death combined and 80 (95% CI 76-84) for all
malignant neoplasms in the workforce as a whole (Table I).

CANCER IN UK ATOMIC ENERGY AUTHORITY EMPLOYEES, 1946-86  617

cd

Cd
0

cq              C>           ON

m         C? m  IC ON w      w  (7S WI as    enIt ON en IT M                       W)    W) C,? 00                    ON      (U
bo                                                  r?      - en tn t-              I?T ON ',O  r- r-                        C)

o                           O?                       C; Cli                 ON wi -4 'i                                                                >

cq

en tn r-       ',O en 00 O's ON c7N CYN c7s IC en en 00 1r) "O  oo en    C)     CD    en    00 11.0 as   tn

00 C14 I-T t--     en en en (= C14 00 IC 0   0        IC!                          C?       cl? -? Cl WI    "C    (21 as

C; ci 6   ci <6 C; <6 <6        p  c,           0     p  0   o           p   (6 C;              C;

Cd
%)                    (7s W)     W)    t-- C?   as 0   M  W  0                                  M   IRt W    c7N    W     en
p"

?Q ,.O 1,0  ,.O    (7N             M  "  C's a?s      ON tn                     r-    en C)     r4    0  C)

(U                                                                                                                                                  010
S.                                                                                                                                                  0  0
Cd

3t                                                                                                                                       >  0

W) IC c7s IC t-- -       r- (7s r-       t- tn r-     en tn     r- IC r-        C)    en 00     C)

r- tn r4 (6 C! W? en C) -                             en       IT C>     W) ON        en WI tn W) r-     C,4    M
Ci        sc             06 r-        "D                 Ci     ei       Ci 0? C?        Ci Ci           C?

C,4          en W) C) W) 00 en     C> ?o 00 1,0      'Rt 00 It     ON en           en    (7,    W) W) en

.C)                        -! n  n  e?        el? O?                             0?                                              oll oo

O?    er! ?q                                                                                                       O? 00   C,3

(D           p c, p 0           p     0            p p (C)         p     p            p p p p            C) p

cd 00

m  m  ON C14 m  w  w  w  tn     W) r4 tn 0   -  C,4      all      'IT 140    CN r-    cn r-                      (U
Q   as    r- 00 r- 00 00 en 1.0 "T W) c7s       en                                                                                +-?-
ed        IZS PI,                                                      . er? Wi Wi O? 0?   r? 111:      fl,       O? W?    Ci C?                       C13

Q         C;                                           6 o 4        6 c,                  C>                                    C) r

. 0

U
as

gz

00 "C      4.)

00

U          14) -Z    00        WI       WI                           WI 00 en              r4     r-    en    r- tn        as           00

P? P.               as       r- M   W  t-                             t-           C!                     r-

o -        -                                                                                                                                       <

?Q  &.                         -: ,6 n  r; m  m  tn m                      00                                                    C14 en
Cd                                                                                                                         C?

r..,.        00        r- "P V-) m  6  m                     tm-       en                 C14   en    -A     el? 06 C-4   -1    -- --

M     W                                                                      t- 00

o          Q 11?3        C) r- ON    M  W)     ON       ON           W     C7., W          t-     en           00          ON     "t

cn                   -                                                                                                                  r-

WI    WI        M                                                     tn en    tn           C,4    C,4      eq       cd

Cd     -St                              (7, t- c m   tn 'r-    t-    t- W  M      r-    r- (7N       W  tn    1,0 tn W) r- C>           00 C>

oo 00                                                              W) en

r- 1,0 a, 10 tn      ',,O r-    m  (7s   IRt w  ON w                  en 00        r- C,,   en           r- oo

0 0 P

Cd                                                      00

+-- .Z -(:3                                                                                                                             06 06

10          U. %)                                                                                                                                   -

as en WI ?,o    W) -                                     C>        r- C)    C)     IC       C",       a,           C14 110  C>

M  t- (=>  -    -  1?0 'It                   ON          rI                 ?o     en    It ON ?,o en --:          0? (7-1
Cd         Q  )-,,      ?=     ,  - -  C) ,-; "6 C!  00 00 ON er) w  tn        m  06 r-                                                    "  en

%)              tn ,   (=) -                                                    r-? o6    o6    tri       o? m

-     en 00           en 1,

'IO 11, en                   C> W)        q- C!    en              4     r-           ri                   v   cd

cli                --tn     --               --    --           --                      w

W  'IT         RON W  ---                "   --    "         CN    CA    ?,o r- -  -  -               r4

Cd                                   M  tn M   Os M     r-     M  M  'O    00 tn 'IT ON    en           r4 'T     Z     ''en               C4 ?=   CA,

C14 en

0  Cd                                M  Ch M   W  en C14 "it   oo ?o r-    'IT en r-       tn r-           en        W) 0? 'IO C>          "O ?o      C13

w  ?-o r- ITt w  r- 1?0 r- (7N r- r- r- 00 tn C?) 00  00 r-     00 r-    "C          (7) M  en C4,)

C13
Cd

00                                                                                                                    en

'IT                                                                                                                   r-   W  Cfs

00                                                                                                    wi   0
4;           4:      00 t-           ri        00                    en rl IC                           00                 00

00                   00        r-           W  W                  M  tn M  'Rt a-, tn   oo M                                            r4 O    cd

ta      -        r-          C.                                                     C>    C?              0? "II:

,; r?: I    . W) NO r4  .  .  .  .  . ON                                                   tn r-

olc?  cid  I?ij   C %)                                                                         C)                        t--  ON

C,4 r- O? W) ?,o (7, cq (7, 00 t- r- 00     en        as

00                           OC) r4 "  r- r-       M            00    ---M         tn           en (7s
64      Q -Q         (=     --C,4                                                          --           ---

-- Q   (Z        M   ?? M            tn  - --- -- t,                                -                                                    cq3
t2o                   WI                                    -- --     It as       IRt                    e-F)  r4 00     W) C>    00     (o W?   1.

4                                               r-       M      -  ?,o

(U

W) 00 W) r- C)     M     tn 0   M  C14 ,It M  tn M  r- C,, I'D  11*      W) C14 ON    00 all C14 1.0 a,-,      tl- 00   S. 0
00 00 1%0 t- C) as r- IC ON <D r- r- t-- ?-c r- (ON r- 00 00 r-    r--          r- 00    as CD ,C r-               r- t--  0

N?                                                                                                                                          cd

Cd

Cd                                                                                                                                                     0

Cd -0
0                                                                                                                                                      *', -CZ

ON                                                                                                                            C,3
(U                          Nt                                       en                                                                                   >

cts

00

C)                  V

E

CIS                                                                  C14                                                                            o  C6 cn

0

>                  r4                                Cd C13

CZ                                                                                                                                C)

C14
Cd
C,3                   t3     E

Q                                            C14                                                                                           44

C13                       =1                                r. C7.1   C13      C,3       0     en                      1.

(U                                          00     S.           qj         (=>                      0      co
U                                                                     t.                                                   C,4 00                         t:

O                                                 C, -, 5   U

-21             en                                                                                              t3            (U 0

W)     a            00                                                             C,3                        rA

C4..                                                                        ON     U            U                    (ON  '... .0

Q               .-    co                                                           0               E                             E
T                                       1-1                           0                 =   0     1. -                                   ..   (ON   0  0

tn              z            2               00              U                            co   ct (U     C:4,(7N
C13                                  CA 10     -            4-    -, 0   1        t3       (U 0 CD Q          (7N

CA r           I--,   0   X                                                             C13

cd 4n .0                          tn

co            t3                                      -               cd -., =    w     -o (7, 0? - (7" M   m  -      C3             4   ?l

00-   .6-1                                         0 -   as                          <D     C>

>     1-1    0  .-             Cd        Cl 'IT 00 en   so r. 00 00                      -W     E                      Cd
co                       >            >           00     oo cq3 -  -                     00.                   Cl

cd    r               ed     =1       E                                               0.0   0   E  E

C3                 E  C's                                      (U     >, Co                           cd cd 0

co                                             cd        00 v         0                                           . o

C13                                                            00           S.                                    ci

cn                                                                                                     S? 00                 en

E  8  0      'd bo    0  U   Cd    r.

Zs --z                                              C:     cd    cd                                                                        r4

C's cd                U.     >  0     cl

t3 OC)    ZZ,           E  C'd 0  >  ,  ,  m  wo  o  o              6.    -         w           ZZ C? 0    0     0

Q  Z-      14? m  0     En .5 04    O. Z   0-4 m  0  U     m     0  a.     m        m            'T "-, Z  X      -?         1121,       E v

618    P. FRASER et al.

At individual cancer sites, SMRs for the oesophagus, stom-
ach, rectum, bronchus and lung, bladder, and brain, were all
significantly low. SMRs for testicular cancer, thryoid cancer,
leukaemia and non-Hodgkin's lymphoma were above 100 but
only that for testicular cancer (SMR  176, 95% CI 96-295)
approached statistical significance (P  0.07). This was large-
ly explained by increased mortality from testicular cancer at
Harwell (with Culham and London) (SMR = 192, 95%, CI
92-353, P = 0.08 based on ten deaths).

For employees with a radiation record, SMRs for all
malignant neoplasms and cancers of the oesophagus, stomach,
bronchus and lung, were significantly low. Additionally
SMRs for breast cancer (SMR = 39, 95% CI 14-85), Hodg-
kin's disease (SMR = 39, 95% CI 11 -99), and multiple mye-
loma (SMR = 36, 95% CI 10-92) were significantly below
100. While prostatic cancer mortality was not raised overall
in employees with a radiation record (SMR = 87, 95% CI
63-118), it was increased in radiation workers at Winfrith
(SMR = 245, 95% CI 134-411, P = 0.005 based on 14
deaths). By contrast, the SMR for prostatic cancer was 69
(95% CI 44-102) at Harwell (with Culham and London) and
66 (95% CI 21-153) at Dounreay. Mortality from prostatic
cancer was higher at younger ages in men with a radiation
record; at ages 45-54 years mortality was increased 3-fold
relative to national rates (SMR = 354, 95% CI 130-771,
P = 0.02 based on six deaths). The SMR at ages 55-64 was
83 (95% CI 36-164 based on eight deaths) and 88 (95% CI
52-139 based on 18 deaths) at ages 65-74. The SMR for
testicular cancer in all men with a radiation record was 171
(95% CI 78-325 based on nine deaths) and 202 (95% CI
55-217 based on four deaths) at Harwell (with Culham and
London) alone.

For employees without a radiation record, no SMR was
significantly increased. Significant deficits were apparent how-
ever for all malignant neoplasms, cancers of the bronchus
and lung, uterus and bladder.

Mortality of employees with radiation records compared with
other employees: rate ratios

When death rates for employees with a radiation record were
compared with rates for other employees using rate ratios
there were few significant differences (Table I). When no lag
was assumed, rate ratios were significantly low for all causes
of death combined (RR = 0.94, 95% CI 0.88-0.99) and
breast cancer (RR = 0.32, 95% CI 0.14-0.77). A 4-fold
excess of cancer of the uterus (including the cervix uteri) in
women with a radiation record was observed (RR = 4.55,
95% CI 1.20-16.66, P = 0.008). Confidence intervals for all
other rate ratios embraced 1.00, including those for prostatic
cancer (RR = 0.81, 95% CI 0.48-1.35) and testicular cancer
(RR = 0.92, 95% CI 0.34-2.07).

Rate ratios were recalculated lagging exposure by 2 years
for leukaemia and 10 years for other causes of death (Table
I). The 4-fold excess of cancer of the uterus in women with a
radiation record persisted (RR = 4.28, 95% CI 1.03-19.97,
P = 0.02). The deficit of breast cancer was also apparent in
the lagged analyses, giving a rate ratio of 0.33 (95% CI
0.13-0.84, P= 0.008) for both sexes combined and, for
women alone, a rate ratio of 0.36 (95% CI 0.14-0.91,
P= 0.02). The lagged analyses revealed a 2-fold excess of
cancers of the bladder and urinary organs (excluding kidney)
or borderline significance (RR = 1.98, 95% CI 0.98-3.97,
P = 0.05) in employees with a radiation record. The rate
ratio for prostatic cancer was 0.84 (95% CI 0.50-1.41) and
0.92 (95% CI 0.55-1.54) with 5 and 10-year lags respectively.

Mortality and level of cumulative external radiation exposure

Table II shows mortality from selected causes of death
among workers with a radiation record according to cumula-
tive external whole body radiation exposure. In the unlagged
mortality analysis in men, prostatic cancer alone showed a
positive trend in mortality with increasing exposure (X2 for
trend = 6.12, P = 0.01), the trend being most evident for

employees at Harwell (with Culham and London) (X2 for
trend = 5.11 ,P = 0.02). The trends persisted when exposures
were lagged by 5 years (X2 for trend = 4.39, P = 0.04 overall
and 6.45, P = 0.01 at Harwell) but were less apparent with a
10 year lag (X2 for trend = 1.65, P = 0.20 overall and 3.70,
P = 0.05 at Harwell). Contrary to the findings in the previous
analysis (Beral et al., 1985), there was no significant trend in
prostatic cancer mortality at Winfrith (X2 for trend = 0.04,
P= 0.8).

The unlagged analysis of mortality in women showed a
trend for all causes of death (X2 for trend = 4.52, P=  0.03)
and all malignant neoplasms (X2 for trend =  5.69, P=  0.02),
the main components of which were cancer of the uterus (X2
for trend = 5.11, P = 0.02) and cancer of the bronchus and
lung (X2 for trend = 4.14, P = 0.04). These site-specific trends
were based on very few deaths - eight cancers of the uterus
(two cervix, five body, one part unspecified) and five cancers
of the bronchus and lung. When P-values for these sites were
checked by simulation, the values obtained were similar
(P = 0.04 and P = 0.01 respectively). Though the significant
trend for all malignant neoplasms was lost in the lagged
analysis (X2 for trend = 2.66, P = 0.1), the trends for the two
specific cancer sites persisted when exposures were lagged by
10 years (X2 for trend = 5.10, P= 0.02 for uterus and x2 =
8.80, P = 0.003 for bronchus and lung). Again the simulated
P-values for these two cancers were similar (P = 0.04 and
P = 0.003 respectively). There were no other statistically
significant trends in site-specific cancer mortality with in-
creasing exposure to radiation from either lagged or unlagged
analyses.

Mortality and monitoring for internal exposure to
radionuclides

Mortality in workers monitored for possible internal expo-
sure to specific radionuclides was examined by calculating
SMRs for comparison with national rates and RRs for com-
parison with other workers who had a radiation record but
were not monitored for exposure to that particular radionuc-
lide (Table III). When no lag was assumed, mortality from
all malignant neoplasms in workers monitored for exposure
to any radionuclide was below national rates (SMR = 80,
95% CI 70-91) but simlar to that of workers with a radia-
tion record who were not monitored for radionuclide expo-
sure (RR = 1.05, 95% CI 0.90-1.24).

Mortality from prostatic cancer was raised nearly 3-fold in
men monitored for exposure to tritium (SMR = 282, 95% CI
113-580, P=0.03; RR= 2.85, 95% CI 1.17-6.95). At Win-
frith, the SMR of 689 (95% CI 188-1763) though based on
only four deaths from prostatic cancer in tritium-monitored
workers was highly significant (P = 0.006) as was the SMR
of 345 (95% CI 139-710) associated with other unspecified
radionuclides (P = 0.01 based on seven deaths). The rate
ratio for prostatic cancer in tritium-monitored workers re-
mained elevated when exposures were lagged by 5 years
(RR = 3.39, 95% CI 1.40-8.24) but not with a 10-year lag
(RR= 1.75, 95% CI 0.50-6.10).

Mortality from cancer of the uterus was raised in women
monitored for exposure to any radionuclide by comparison
with other radiation workers (RR = 17.99, 95% CI 2.14-
290.14) but based on only two deaths. There were no deaths
from cancers of the bronchus or lung in women monitored
for radionuclide exposure. A 2-fold excess mortality from
cancers of ill-defined and secondary sites was observed in
radiation workers monitored for radionuclide exposure com-
pared to other radiation workers (RR= 1.95, 95% CI 1.01-
3.77). This was largely confined to workers monitored for

radionuclides other than tritium or plutonium (RR = 2.39,
95% CI 1.22-4.69).

In order to investigate further the effects of internal and
external radiation exposure, the trends in prostatic cancer
mortality were re-examined after stratification for radionu-
clide exposure (Table IV). Rate ratios for prostatic cancer
mortlaity increased with increasing cumulative whole body
exposure in men monitored for exposure to any radionuclide

CANCER IN UK ATOMIC ENERGY AUTHORITY EMPLOYEES, 1946-86                                               619

0

+ +                  + +          +     +           +     + +                                  + + +

Cd

tn ON                                            00 C? C14   00                 00

Z  0? r- '.0   4  6  oo -                                                   8D I      I",  (= '_ -           E

0                                                                                                                                   (U

06 6  6  wi                                                           8  (6 "i

+ +                  + + + + + + + +                                        + +

>

(U

en as ?o    "D oo oo oo '?t cn o        eq       00       C'4 en      "C    "D           cq    00 "o

en Co                             WI                                  C) It              en kn

4 8         c? "6 6     6 6       6 6 6       6     6 6

Cd

0

X:    kn a, cn 00        W) W) "D "D 00 W) C'4 ON    C14 't  't                                    W)

110   C14   en    C14   "   -  -          Cd
o                                                                                                                     as -          04

(A                                                                                                                 C14 C14          0

(U

(U

00 r-

(U

00

00    "o r-   'so c?    (O r-     C) W  al?     'so          "o    W     M  W) -      tn   I",

0?    06 C'?'-! 0? m    ln'n m  0

c?          r-?   vi 6  r'i    c?    W)

C's      -'so M   M  M  -  -   -                 VI) C14  en <D 't    't    en C>              C'4 en C'4  00
(u              1-1       1-1 ll? '_? '.? '_? '_? '.? '.?         1-1 1-1  1-11 1-1 I---I  1-1  I--- I 1-1      1-1 1-1 I.-, C14 ON

(s, WI r- 't IC eq en 00     tn ON a-,     'IO a-,   00 C14   aN     00    r-    en                      oo (71 c?  ed

<D C14      Q  r- r-        ON "o C) 0  CD W)    en <D    clq   r-    in     a's  en              ON r- Z'
A      ci 6                        ci 6  (6 c? c?                                                           t

.00

0

?o 00

C>

W     4n tl-         tn tn                    M  (2N               CA       'so tn    C>       C'4

Cd                  00 C)       00 ?o IC 00           C'4   "D ?.o   V- t-    tn M  W     r.:                            00

CD                         C4 (6 'i           C4 6  vi    c?          cq
ON                   WI -_ -_ -_                          --- --- ---       --- --- ---        en r4 -_
00 M     t- IC M     M     C> 0                           en    tn          't C14 't    C14   C14 en 't

00
C'4   ?p          tn V'?       C? 'IO   c7_1        cn    00 00 'IO   t-    oo c, tn
r- 00 r- O's 00 tn C'4 ON C>   r- (= oo       oo tn    -  C14 C)   tn    en tn              ON 0  W)

00 0                         6  6  C5 6      vi 6  ci 4  r.? 6  6    6  6     -4 "i Z;    -4

cd

C's
tn                                                                                             00 ?C

ci                                                                                             ON 00

r- 00                                                        r-                        . 0; cn

"t CD t- '-N   '-,                "-,                  00    en                                       (U

"C en "6 C4 V? CD 06 00 rI r-- 0  en t'-      00 -     en S  C'i         00 S  ?o
(U       _?%                                                                                 r- C)

__ r-          r, -? tn        00 00 "T r- tn    -    r-  V-? 't a;           .     . '=?

C'4               r-                             r-: C4   4     6           tn                 a? CA

cd          -tz     t-                ---                            -_ -_    -_          W)

00 IC V4       ?.o IC CD Q    C) m                                                             00 r-

00
en             'C r               00   110    C?    rl Q  en    00
r? en 00    00 c. 0  c, q  Zq       a, en  tn       Z  "t WI

r- C>
qa                                                                                                               en

co
0

W)                                                                                                              cl

,Q       p        W) w     0,41 w   w     W) cn     C14 (7s 00        en aN       C14         0?

0? 'IR      r'?   4  'q r? 17, 1=    m        "* C)    tn -     -        C> "T             en     00         E

W) tn -6    c)                       c?       ci             4     C;

W) M                              m                       W)

W)

>     E                                                                                                                        C14

r-    oo          as 00    (71, 00  I",    C)    ?o      'IO 00    r-    oo oo ?o         5
O? W)    0" ON C'4 ON r- <D    C'4 <D C> t-- t?              CD m     C'4                      a, C., a?

c, C;       C5    6     6  6   C?? 6   6   C; 6              C5                C4 6      6     6  C; 6  ?ro-

00

C14

ON tl- ?o all                                                Ch                          00

aN       oo 'l- r- oo cn               cn                       W)    C14   tn           C14                   (U
73                                              "  kn tn    m                                                   Fl     C!

C14   r- v.:            a4?'                                 00 "6

en                      tl- as 110   r14              '?t -     r-:    c? -

--_en     . "                                                         C14                        -0-N
cq3              en ---"D

00                      cn en C'i W), en'  tn      tn

4.                                                 m            W)       W)

(U                                                                                                                       cn

0 >

w     -     r- W) ,D "o    00        (ON  IC     -     en C'4 r-   r-    oo r- CD     W)
a? c7" -    w   -   r- m   a-, 0? m   w   ?'c tn w   a-,  0   all  0? C? Iso  00  "C 110 C>  C14  a-, (7-, a,  tn

c;    c; c; c; c; C5       C;     C; 6    6   6     6     6              c  c  'C'

Cd 0                                                                                                                                >

Ucl                                                                                                                                 C)

>

(0

X                C14

36?                                          C)
0            00

o1v
d-I                                                                            S.

cl

E                                                                                       93    0

00                    C>

(L)                                           CL.  "  oo

en                                            en              E       (=> U
r.0                                                                           C-4    0 -z              CA

0                    21.                                                             u

Q                                                          0

qw                                                                                            as

lr?                   (U   00                    0         w                                              0
ce             -Z                U                                    u     >                       o     u  u     ON ON

r-                          0  X     E.    -,'O                     Q  x              cd

u                             -u elx    u

3z      14n""T W)                                      0    en C               >' '-, C)                w  E

0           oo              =    a-, el       u '-N

VI   cl    cj                            rl?                            00

'Tr 00            00 00    (7.,            to,                  -  Z.,

r-                                                E                       cd=     C13

'O -         .Z "  V  -                           (U cj  cj

Cd =  =     (A0                              1-1 r                             E      14i

C13                                                    0

>' C'31                                 ? cr?

rn          (U 00                   6            (U (U

C's U:3     U                                                            cd C13                  el
0  EOa      U  r-              >' C13     oo       ON Cd              5     -w -w (o 1:3 C', 1:3  o

0           0                                                      cq

>  0     ct3                         0 '-.' :3                        U

Zz

o                                    z        0 U

620    P. FRASER et al.

0)

~CA

0 -i

~~  ~ ~ -~~~--ri~C4',Cr 00  ti  * N~ 00  00 _. W)  ON -   00
0        ~~.                    r~ir~-   -r-in-oo  CO

W  -  -    -  o   W tn(N      C>r-r--

COd

0)  ~ ~ ~ ~  0                            0 IT  COW

~~~~  -~~~~~~~~  N   ~ ~ ~ ~ ~ r  4  qC    Clo  -d

<D 0 oo O a-  !0 tn (C  0  t

CO                                          0 ~~~~~~~;6 p  C ~ 0)

-~~~~~~t  'tenq 0  e) a 10r  00 ~ 0   'IT  C t-N  (N C  00(  0oo
o  ~~~~~~~~~~N -    00 fl  00  ON  ON 0O0 00  000

M "'.-          - 0  (N   0  00  N.-.C

x                      00~~~~~~(  00  ! N I N   o

-o  ~~~~~~     'rb-~tn(7  -  -D  r  -  -       COt  C   W

0       _~~~~~~~~~~~~~~~~~~~~~~~~~~~~~100

CD o 1 as 0o  enC 'C -.

-O ON~o'Cr'IO~

q6)~~~~~~~~~~~~~~~~~~~~~~~~~~~~~~~~~~~~~~~~0

0  NrC)( en en0O.CO.0N  00  'Ct  On  N0  O'-Mtr  0 ~ I

04 -~~~                        00ON00O -   N  o

O-OOO-~~~~-O  0-  -  (N ~~~~6  6C5 C6  6  (6 6  0

CO                                       .~~~~~~~~~~,  ,O  4  nC

~~~~~~~~~~ r-~~~~~~~~~~~~~~~~~~~~~~~~C c

N D000  .0  W~  C4  0~ Ne N  00  -NrI'

0)~~~~~~~~~~0

0)C         m  a   06  tN

U,     (N0\0(NN0~  ~(9C> 'IO c  0 -  en  ;(N1  00 C)C

C4  Ci Ci  C-       0 01

(NN-r- 0   tn  (N  ' -  c'00  Cf - n
IVIen e  Iq  It  Iq  en R  6   I

00~~~0N N~~N   00-~C                  0 N Ne

0)~~~~~~~~~~~~~~~~~~~~~~~~~~~~~~~)t

CO~~~~~~~~~~~~~~~~~~~~~~~~~~~~~~~~~~~~~~~~~~~~C

ON  ) m 01%It  n  (N  - 'I            CO)

-     ~~~~~~~~ON  0) _  ( 0         C
t as 0Nr0 'CtNr  -'"N  (N N  -"   00~ 'it-  0

Co                 000           I  IC..I  I  ii

I  liii  ~~~~~~~  I  II  I  N~~~~~  00 trCON  ON  ~ ..,0
(n  N  r "O  aN,,N  00  00 W  t  r

~~~  ~~~~ o5 n  6  6

~~~ (N(N  N~~~~~~~~~~6f  CDC> C;C

k ~~'- 6~~66666C>oC>C>  66  6 6 --0-   o6-

-o  3..N  (~~~~~n ( n ---.   -  c N

CO  ~ ~   ~   ~     -  -- ---  - C   (N  ~t,  (cO( C ' ON

C3                              0d

0 ~~~~~~~  (N  CO~ ~ ~ ~~~~ONC

r-O -m4  m   m  o 00 en(N en0 .   00  C4

eq en    -   C14     O

ON 0
C4 'O                        X

0                 'U C-4

I--, >,,-, 0 )-t - C)

V                        a-, (71 0C4 CZ 5, z6? _w

00          00 00                        C4

,=> r= C's 00

It                                            ON    a-, ts C) 00             C)    CN

a, 00     +_1 1-1 05W -  "? 00 M                     Z     -Z)

C>                                                                         CN    all

1    en

I=, Cd                            >,m                                  "'t                        (U

00             tn     6 X e                                        "V

4-A .-   00

'10-                                        (=                  bo>

rq    cq           C4    Q

cd                                :3 :3='-,                        0

C,3   C,3         (L)                                      0tu     -._ -  --- =0

"T Z,              0. m     P. F.                       ,,t   Z            0_?   "T    "Tz    E

CANCER IN UK ATOMIC ENERGY AUTHORITY EMPLOYEES, 1946-86  621

(x2 for trend = 6.46, P = 0.01) but not in radiation workers
who were not monitored for radionuclide exposure (X2 for
trend = 0.01, P = 0.9). Prostatic cancer mortality increased
with increasing external exposure in men monitored for expo-
sure to tritium (x2 for trend = 4.24, P = 0.04) but not in
radiation workers who were not monitored for this radionu-
'0                                                      clide (X2 for trend = 0.31, P = 0.6). The trend in rate ratios

Cd        o4for tritium-monitored men was based on only seven deaths

u    ->                      Qall of which occurred in the highest category of external

--+  +   +    +   +    +   I

o- |      -   - xi | +  + | ;  + | +  +  exposure (SMR = 531, 95% Cl 214-1094); the P-value for

00  ON   ON  co   -    -   1   0

v 0                   .o  c-  en    e           trend obtained by simulation (P = 0.06) was similar to that

"Q  9          0,       0    - o  _  o  _  o  N  o based on the chi-squared statistic. Prostatic cancer mortality
oX also increased with increasing whole body exposure in men

monitored for exposure to radionuclides (unspecified) other
o                           z                           > " " " " than tritium or plutonium (x2 for trend = 5.97, P = 0.02) but

+   ++   +   +      +    +      I

not irradiation workers who were not monitored for other
'IT |> E- ?0 < |o  -| ; ^ |>  t |  es [ radionuclides (X2 for trend = 0.22, P = 0.6). These trends
:0  0 o      0    0 o  N <     0 o      however are not independent as more than half of the
E                                                      radionuclide-monitored workers were monitored for exposure

to more than one radionuclide and thus contribute to more
2 ^                              than one category in Table IV.

>Z o1 _  _        I-,          There was no association between prostatic cancer mortal-

? <D CA                   WI                           ity and monitoring for plutonium exposure. The significant
-     0 _   ___,   o trend in radiation workers who were not monitored for

plutonium is due to multiple monitoring; seven of the eight
0                                                      men in the highest exposure category, whilst not monitored

for plutonium, were monitored for exposure to tritium and/
Q0        0 G;  -  ?  o  ?  ?  ?or other radionuclides. There were no significant trends when
e        0   w,   _        N              these analyses were repeated incorporating a 10-year lag
en |                                  (Table IV).

CA       X < ^ o _ _    o > _ , _ > o t o ofi _Only two deaths in radionuclide-monitored women pre-

o0 D   zcluded useful analyses of the trends in women for cancer of
o. ^  >the uterus after stratification for radionuclide exposure.

0~~~~~

~~  .~~  e~~~  ~~-  -   ~00  ON  Cl 00  e'n

X t                 6b   -          0 ovi 0x          Risk estimates

IQ        0   0 ?   t0          Risk estimates for leukaemia were negative regardless of the

co 00           00.0 .~-

X;   _0    0           -   0 - -    0 o >    lag period assumed (Table V). The estimate of -4 leukaemia

deaths per 104 person-years per Sv contrasts with an equiva-
lent figure of +0.4 deaths (95% CI -3.2 to +9.9) found
S        E0'                                           previously (Inskip et al., 1987). For all malignant neoplasms

U                       o                     except leukaemia, risk estimates were positive although con-

ri~~~o  0~00                00

0 C4 .              6 o  ~   ci o ~   -    ' _       fidence intervals suggest that a wide range of values, both
0  lo -  _)    _- z      .       positive and negative, are compatible with the data. The
Q E' o  I o  ot o   I o  x o   ro N > O  estimates of +20 deaths per 104 person-years per Sv for all
.0                                                     malignant neoplasms except leukaemia compares with a

figure of + 17 deaths derived from previous analyses (Inskip
et al., 1987).

o           ~~        ~       ~~~~ON  cl

KK |           b                                      ^ | O -   ..  Cancer registrations compared with national rates:

~00~-                 0 o o  _  o  _  e>, t o.  standardised registration rates

C i9r-~ ~      .rw9

A total 1,699 neoplasms (ICD 140-239) registered during

o                                                      1971-84 in 1,657 individuals were analysed. When person-
X   |        1:4  04 X 4   04 X 1 2  =  1 > X > X 1 a:  X years-at-risk as calculated for the mortality analyses were

4 u: X    4  04 En m X  X co X co X4  04 X 4 coX used the standardised registration ratio (SRR) in the work-

_4 _  force as a whole was 88 (95% CI 84-93) based on 1,632

0                                    C>~~~~~~~~

3--               r                              o     registrations (i.e. excluding 67 cancers first identified through

0

;         r -                   2             v     a death certificate in 1971-73). The SRR was 87 (95%  CI
X    0 r   E  = N.  82-93) for workers with a radiation record and 90 (95% CI
.             =0  e5 E                                 83-96) for non-monitored workers. Site-specific SRRs were
E~ r     .2 : ~ ::  g  X  ?  also calculated by censoring person-years-at-risk and registra-
r   ;  <  .:  ;  o  v  Q   ?    V    tions at the date of first registration for cancer at that site.
2                 0 o     .0 o     . v   -       CA..  This increased the SRRs by 4% on average, the overall SRR

v:4 o  e  o  t                       being 92 (95% CI 88-97) based on 1,592 registrations. The

0       latter method for calculating person-year-at-risk was used in
o     o    o  0    0      0   00 o      all subsequent site-specific analyses on the assumption that
0 .0 E  .=  E  .= E  .          an individual diagnosed with cancer is unlikely to be at risk
"  |   1 :E  ?  1 S  z?  1 :E z  z o  o;  of another primary cancer at the same site.

Registrations for employees with radiation records compared
with other employees: rate ratios

Fatal cancers dominante the cancer registrations for most
cancer sites and analyses of fatal cancers have been reported

622    P. FRASER et al.

Table V Risk estimatesa (with 95% confidence limits) for leukaemia
and all malignant neoplasms except leukaemia obtained from additive
relative risk models using lag periods of 2 years for leukaemia and 10

years for all malignant neoplasms except leukaemia

Absolute excess risk
Excess relative risk  (per 10i person years

(per Sv)             per Sv)b
Leukaemia (ICD8 204-208)

Unlagged           -4.1 (-5.7, +2.9)    - 3.5 (-5.4, + 1.8)
Lagged             -4.2 (-5.7, +2.6)    - 3.9 (-5.9, + 1.7)
All malignant neoplasms except
leukaemia (ICD8 140 -203, 209)

Unlagged           +1.2 (-0.4, +3.1)  +19.9(-6.4, +48.0)
Lagged             +0.8 (-1.0, +3.1)  +20.3(-26.0, +71.1)
aAdjusted for age, sex, calendar period, establishment and social class.
b Equivalent to 106 person-years per 10 mSv used in previous analyses
(Inskip et al., 1987).

in detail in Tables I to IV. The registration analyses reported
here were restricted therefore to cancer sites where previous
analyses have suggested a relationship between mortality or
cancer incidence and radiation exposure, and sites which
carry a better prognosis where an examination of cancer
registrations might be expected to provide additional inform-
ation. The extent to which fatal cancers dominated the
cancer registrations even for these selected sites is shown in
Table VI; only 429 (27%) individuals with a malignant neo-
plasm registered in 1971-84 were alive at the end of the
study period in December 1986.

When registration rates for employees with a radiation
record were compared with rates for other employees there
were no significantly raised ratios in either lagged or unlag-
ged analyses (Table VI). In the latter, the rate ratio of 0.38
(95% CI 0.15-0.98) for testicular cancer was significantly
low (P = 0.05). There was no evidence of excess registrations

Discussion

Cancer mortality

This report describes mortality from all causes, and cancer in
particular, among 39,718 employees of the UKAEA over a
41-year period from 1946 to 1986. The analyses are based on
a total of 5,509 deaths, representing an increase of 63% over

the number of deaths included in our first report which
covered 34 years from 1946 to 1979 (Beral et al., 1985). This
substantial increase in material permitted more detailed
analyses to be carried out for site-specific cancer sites than
previously. Comparisons have been made with mortality in
the general population of England and Wales, between em-
ployees with a radiation record and other employees, and
between groups of radiation workers accumulating different
levels of external radiation exposure. Mortality has also been
examined in workers monitored for internal exposure to
tritium, plutonium and other unspecified radionuclides. The
of non-fatal breast cancer among women with a radiation
record (not shown) as had been the case in the analysis
reported in our previous paper (Inskip et al., 1987).

Cancer registrations and level of cumulative external radiation
exposure

In unlagged but not lagged analyses there was a significant
trend in prostatic cancer registrations with increasing expo-
sure overall (x2 for trend = 6.34, P = 0.01) and at Winfrith
alone (X2 for trend = 5.52, P = 0.02). These trends are based
on 74 and 23 registrations respectively. In women the unlag-

ged analyses showed trends for all malignant neoplasms (X2

for trend = 5.43, P = 0.02 based on 61 registrations) and for
invasive cancer of the uterus (X2 for trend = 4.83, P = 0.03
based on eight registrations) which persisted in the lagged
analyses. The uterine cancers were all fatal and thus the
findings largely replicate those of the mortality analysis.

In our previous analysis of non-fatal cancers in relation to
cumulative whole body exposure (Inskip et al., 1987) the
trends for skin cancer and bladder cancer were suggestive of
an association (X2 = 3.65, P = 0.06 and x2 = 3.57, P = 0.06
respectively). These findings were not replicated when the
trends for these two sites were re-examined in subjects
registered in 1971-84 who were still alive at the end of the
study period in December 1986. The chi-squared trend statis-
tic for skin cancer was 0.12 (P = 0.7) in unlagged analyses
and 0.03 (P = 0.9) in lagged analyses based on 71 registra-
tions. The corresponding statistics for bladder cancer were
1.92 (P = 0.2) and 0.97 (P = 0.3) based on 25 registrations.
Non-fatal cancers of the prostate were also examined in
relation to cumulative whole body exposure but there was no
evidence of an association (X2 (unlagged) = 1.91, P = 0.3 and
x2 (lagged)= 0.17, P = 0.7, based on 21 registrations).

Table VI Percentage survival at end of study period and registration rate ratios for selected sites for
employees with a radiation record compared with other employees, adjusted for age, sex, calendar period,

establishment and social class, 1971 -1984

Cancer site                        All       Survival    Rate ratio (95% confidence interval)
(ICD code (8th revision))      registrations  % (No.)        No lag          10-year lag
All malignant neoplasms

(140-209)                         1594       27 (429)   1.01 (0.90-1.13)  1.07 (0.95-1.20)
Skin (172-173)                      178      72 (128)   1.05 (0.75-1.47)  1.14 (0.82-1.59)
Breast (174)                        135      42 (57)    1.02 (0.64-1.64)  0.97 (0.58-1.62)
Uterus (180-182)                    37       49 (18)    1.44 (0.62-3.37)  1.82 (0.77-4.26)
Cervix in situ (234.0)              25       96 (24)    0.74 (0.17-3.30)  0.97 (0.22-4.35)
Ovary (183)                         28       32 (9)     0.82 (0.27-2.47)  1.00 (0.33-3.03)
Prostate (185)                      105      25 (26)    1.34 (0.85-2.10)  1.43 (0.92-2.22)
Testis (186)                        20       65 (13)    0.38a (0.15-0.98)  0.39 (0.14-1.11)
Bladder (188, 223.3)                94       50 (47)    1.14 (0.73-1.79)  1.15 (0.74-1.78)
Brain and nervous system

(191 -192, 225, 238)b             35       20 (7)     0.89 (0.41-1.92)  0.80 (0.38-1.71)
Thyroid (193)                         7      43 (3)     0.36 (0.06-3.93)  0.54 (0.11-4.21)
All lymphatic and haematopoietic

(200-209)                           119      25 (30)    0.79 (0.53-1.20)  0.85 (0.57-1.29)
Non-Hodgkin's lymphoma

(200, 202)                        45       33 (15)    0.91 (0.45-1.83)  0.85 (0.43-1.67)
Multiple myeloma (203)               13       8 (1)     0.28 (0.06-1.72)  0.36 (0.08-2.08)
Leukaemia (204-208)                 49       16 (8)     0.77 (0.41-1.46)  0.79 (0.42-1.51)
Leukaemia except CLL

(204-208 except 204.1)            36       11 (4)     0.63 (0.29-1.34)  0.65 (0.31-1.40)
All neoplasms (140-239)            1657      29 (475)   1.00 (0.90-1.12)   1.06 (0.95-1.18)

Significance: ap < 0.05. All significance probabilities and confidence intervals have been calculated using
exact methods where observed registrations <20. bIncludes benign and unspecified neoplasms of the
nervous system.

CANCER IN UK ATOMIC ENERGY AUTHORITY EMPLOYEES, 1946-86  623

results will be discussed here within the context of the
previous findings, which have been described fully elsewhere
(Beral et al., 1985; Inskip et al., 1987; Carpenter et al., 1988;
Carpenter et al., 1990).

As previously, overall mortality in the UKAEA workforce
was lower than the national average in England and Wales as
a consequence of health-related selection and other differ-
ences between employed people and the general population
(Carpenter et al., 1990). Cancer mortality remained about
20% below national rates and rate ratios for all malignant
neoplasms indicated no overall difference in cancer mortality
between workers with a radiation record and other employ-
ees. As before, mortality from testicular cancer, thyroid
cancer, leukaemia and non-Hogkin's lymphoma was above
average in the workforce as a whole but only that for tes-
ticular cancer approached statistical significance. Attention
had been drawn previously to increased mortality from tes-
ticular cancer, particularly at Harwell (Beral et al., 1985), but
there was no evidence in these new analyses that this finding
was associated with exposure to radiation.

As in previous analyses, prostatic cancer mortality showed
a clear relation with radiation exposure. There was a statis-
tically significant association with increasing cumulative
whole body exposure although the trend diminished in
strength with longer lags. The pattern of prostatic cancer
mortality across age groups suggests, as before, that the
highest risk (relative to national rates) is in men aged 45-54.
Mortality was significantly raised in men monitored for expo-
sure to tritium, the rates being increased nearly 3-fold in
comparison with national rates and other radiation workers
who were not monitored for tritium exposure. At Winfrith,
where prostatic cancer mortality was significantly increased
in employees with a radiation record, mortality was also
raised in men monitored for exposure to tritium, and to
radionuclides other than tritium and plutonium. Further
examination of the dose-response relationships after strati-
fication for radionuclide exposure indicated that the associa-
tion between prostatic cancer mortality and cumulative whole
body exposure was largely confined to radiation workers who
had also been monitored for radionuclide exposure, notably
tritium and unspecified radionuclides. The highest risks were
in radionuclide-monitored workers who had accumulated at
least 100 mSv external exposure suggesting as before that
men with these dual exposures are most at risk. The findings
for prostatic cancer are now being investigated further in a
case-control study based on UKAEA records to determine if
prostatic cancer can be linked to any particular aspect of the
employment history of the individuals concerned. Particular
attention is being given to radionuclide exposure and the
potential for contamination of the work environment by
radionuclides, chemicals and other substances.

Mortality from cancer of the uterus (including the cervix
uteri) in women with a radiation record was increased 4-fold
by comparison with other employees. As in the previous
report (Beral et al., 1985), cancers of the body of the uterus
made the major contribution to the excess mortality. The
persistent trend in these analyses, whilst based on only eight
deaths, is supportive of an association between mortality
from cancer of the uterus and external exposure to radiation
and warrants further study. Contrary to the findings in the
previous analysis, there was no suggestion of increased
mortality from ovarian cancer.

Although the increase in the average duration of follow-up
from 16 to 22 years allowed for longer latency and greater
statistical power, cancer of the prostate, and possible the
uterus, are the only malignancies which show an association
with radiation exposure in the UKAEA workforce. It is

noteworthy that mortality from multiple myeloma which has
been associated with radiation exposure in the Sellafield
(Smith & Douglas, 1986) and Hanford (Gilbert et al., 1989)
workforces was not associated with radiation exposure in
UKAEA employees. Indeed, the SMR of 36 (95% CI 10-92)
based on four deaths from multiple myeloma in employees
with a radiation record was significantly low by comparison
with national rates (P = 0.03).

Despite the additional 7 years of follow-up yielding a
substantially increased number of cancer deaths, uncertainty
associated with the risk estimates remains large (Table V).
For leukaemia, the data are compatible with decreasing risks
or increases of up to two deaths per 104 person-years per Sv
(or an excess RR of 2.6 per Sv). These risk estimates are very
similar to those obtained for US nuclear industry workers
(Gilbert et al., 1989) but contrast with the positive values
recently provided for adult males from A-bomb survivor data
of 5.0 deaths per 104 person-years per Sv and an excess RR
of 3.7 per Sv (UNSCEAR, 1988). Comparing these latter
estimates with those relating to nuclear industry workers is
questionable because they are likely to be affected by many
factors thought to modify the risk of radiation-induced
leukaemia. These include the type and duration of exposure
and other characteristics of the populations studied. Recent
analysis of a much larger population of UK nuclear industry
workers (Kendall et al., 1992a,b) (of which the current
UKAEA cohorts forms a part) are more appropriate for
comparison. This provided an excess RR estimate for
leukaemia of 4.3 per Sv (90% CI 0.4 to 13.6).

In contrast with data on US workers, the central risk
estimate for all cancers except leukaemia obtained from our
data was positive (excess RR per Sv (lagged) = 0.8, 95% CI
- 1.0 to 3.1). Data from the larger population of UK
workers are also suggestive of a generally positive association
(excess RR for all malignant neoplasms = 0.5 per Sv, 90% CI
-0.1 to 1.2) (Kendall et al., 1992a,b). Estimates of absolute
risk from our data ranged from a decrease of 26 deaths per
i04 person-years per Sv to an increase of about 70. Whilst
these include the value recently recommended by the Interna-
tional Commission on Radiological Protection (ICRP) for
working populations of approximately 14 deaths per 104
person-years per Sv (ICRP, 1990) (assuming a 25-year ex-
pression for lifetime risks), our estimates are not sufficiently
precise to rule out estimates of up to five times larger than
this. To lessen this uncertainty, we have combined the
UKAEA, Atomic Weapons Establishment (Beral et al., 1988)
and Sellafield (Smith & Douglas, 1986) study populations in
a further analysis (the Nuclear Industry Combined Epidemio-
logical Analysis). The combined study population of 75,000
nuclear industry workers will permit the relationship between
exposure to low-level ionising radiation and cancer mortality
to be estimated with greater precision than was possible in
any of the three studies individually.

Cancer morbidity

Because of high case fatality for most cancer sites, the find-
ings for cancer registrations largely replicated those of the
mortality analyses. In particular, trends in registrations of
prostatic cancer and cancer of the uterus with increasing
exposure were apparent. The dose-response relationships re-
ported before for non-fatal skin and bladder cancers (Inskip
et al., 1987) were not replicated here. The previous findings
were based only on cancer registrations in ex-employees who
may not have been typical of the workforce as a whole.
These new results based on cancer registrations in all em-
ployees suggest that the previous findings may have been
biased.

Conclusion

This analysis of a much larger body of material than that
reported in our first analysis of the UKAEA workforce
generally confirms the robustness of the previous findings for
most cancer sites. The association between prostatic cancer
and both internal and external exposure to radiation is still
evident though the dose-response relationship is diminished
in strength. Prostatic cancer is under investigation in a case-
control study within the UKAEA workforce. The association
between cancer of the uterus and external radiation exposure
which has strengthened in this analysis also warrants further
study. There are a number of other statistically significant

624   P. FRASER et al.

results, as would be expected by chance alone when such a
large number of comparisons are made, but no other cancer
sites with consistently exceptional findings. Uncertainty still
surrounds estimates of the increase in cancer risk per unit
dose. Further combined analyses will provide more precise
estimates.

Members of the Epidemiological Monitoring Unit were funded by
the Medical Research Council through contracts held with the
United Kingdom Atomic Energy Authority. We thank members of
these organisations for their support and co-operation. We are par-
ticularly grateful to Len Salmon, Dallas Law and Jean Rose for their
help. We also thank staff of the National Health Service Central
Registers at Southport and Edinburgh, and the Department of Social
Security Records Branch and Information Technology Services
Agency in Newcastle for providing follow-up information. We are
grateful to Evelyn Middleton and Juliet Jain for secretarial assis-
tance.

References

BAILAR, J.C. & EDERER, F. (1964). Significance factors for the ratio

of a Poisson variable to its expectation. Biometrics, 20, 639-643.
BAKER, R.J. & NELDER, J.A. (1982). The GLIM system. Release

3.77. Generalised Linear Interactive Modelling. Oxford: Numerical
Algorithms Group.

BERAL, V., INSKIP, H., FRASER, P., BOOTH, M., COLEMAN, D. &

ROSE, G. (1985). Mortality of employees of the United Kingdom
Atomic Energy Authority, 1946-79. Br. Med. J., 291, 440-447.
BERAL, V., FRASER, P., CARPENTER, L., BOOTH, M., BROWN, A. &

ROSE, G. (1988). Mortality of employees of the Atomic Weapons
Establishment, 1951-82. Br. Med. J., 297, 757-770.

BRESLOW, N.E. & DAY, N.E. (1987). Statistical methods in cancer

research. Vol. 2. The design and analysis of cohort studies. IARC
Scientific Publications, No. 82. Lyon: International Agency for
Research on Cancer.

CARPENTER, L., HARTNELL, J., BROWN, A., BOOTH, M., BERAL, V.

& FRASER, P. (1988). United Kingdom Atomic Energy Authority
Mortality Study: Supplementary Tables. Atomic Energy Research
Establishment R 13007.

CARPENTER, L., BERAL, V., FRASER, P. & BOOTH, M. (1990). Health

related selection and death rates in the United Kingdom Atomic
Energy Authority workforce. Br. J. Ind. Med., 47, 248-258.

FRASER, P., BOOTH, M., BERAL, V., INSKIP, H., FIRSHT, S. & SPEAK,

S. (1985). Collection and validation of data in the United King-
dom Atomic Energy Authority mortality study. Br. Med. J., 291,
435-439.

FRASER, P., MACONOCHIE, N., CARPENTER, L., HIGGINS, C.,

BOOTH, M. & BERAL, V. (In preparation). Mortality and cancer
morbidity in United Kingdom Atomic Energy Authority employ-
ees, 1946-86. Atomic Energy Authority Report.

GILBERT, E.S., FRY, S.A., WIGGS, L.D., VOELZ, G.L., CRAGLE, D.L.

& PETERSEN, G.R. (1989). Analyses of combined mortality data
on workers at the Hanford Site, Oak Ridge National Laboratory,
and Rocky Flats Nuclear Weapons Plant. Radiation Res., 120,
19-35.

GILBERT, E.S. (1989). Issues in analysing the effects of occupational

exposures to low levels of radiation. Statis. Med., 8, 173-187.
ICRP (1990). Recommendations of the International Comission on

Radiological Protection, Publication 60. International Commission
on Radiological Protection. Pergamon Press: Oxford.

INSKIP, H., BERAL, V., FRASER, P., BOOTH, M., COLEMAN, D. &

BROWN, A. (1987). Further assessment of the effects of occupa-
tional radiation exposure in the United Kingdom Atomic Energy
Authority mortality study. Br. J. Ind. Med., 44, 149-160.

KENDALL, G.M., MUIRHEAD, C.R., MACGIBBON, B.H., O'HAGAN,

J.A., CONQUEST, A.J., GOODHILL, A.A., BUTLAND, B.K., FELL,
T.P., JACKSON, D.A., WEBB, M.A., HAYLOCK, R.G.E., THOMAS,
J.M. & SILK, T.J. (1992a). First analysis of the National Registry
for Radiation Workers: Occupational exposure to ionising radiation
and mortality. NRPB-R251.

KENDALL, G.M., MUIRHEAD, C.R., MACGIBBON, B.H., O'HAGAN,

J.A., CONQUEST, A.J., GOODHILL, A.A., BUTLAND, B.K., FELL,
T.P., JACKSON, D.A., WEBB, M.A., HAYLOCK, R.G.E., THOMAS,
J.M. & SILK, T.J. (1992b). Mortality and occupational exposure to
radiation: first analysis of the National Registry for Radiation
Workers. Br. Med. J., 304, 220-225.

SMITH, P.G. & DOUGLAS, A.J. (1986). Mortality of workers at the

Sellafield plant of British Nuclear Fuels. Br. Med. J., 293,
845-854.

UNSCEAR (1988). Sources, effects and risks of ionizing radiation.

Annex F. Radiation Carcinogenesis in Man. United Nations
Scientific Committee on the Effects of Atomic Radiation. E. 88.
IX. 7, United Nations New York.

				


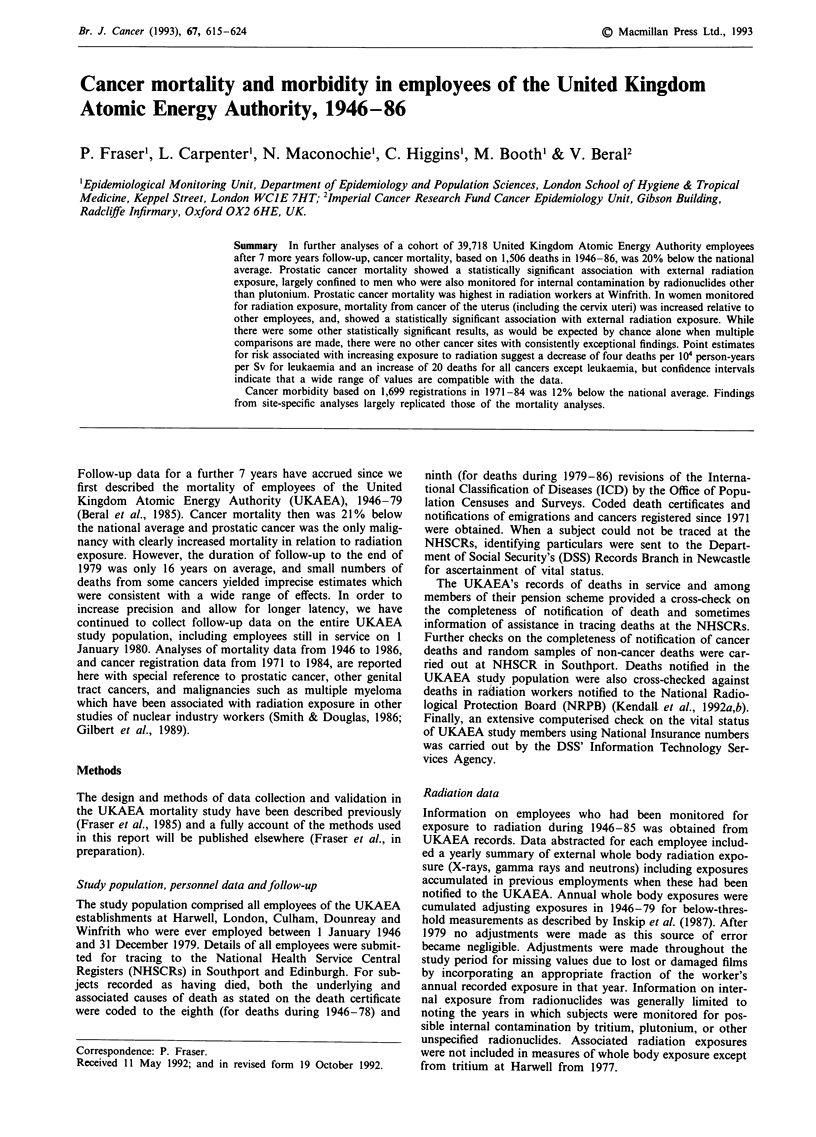

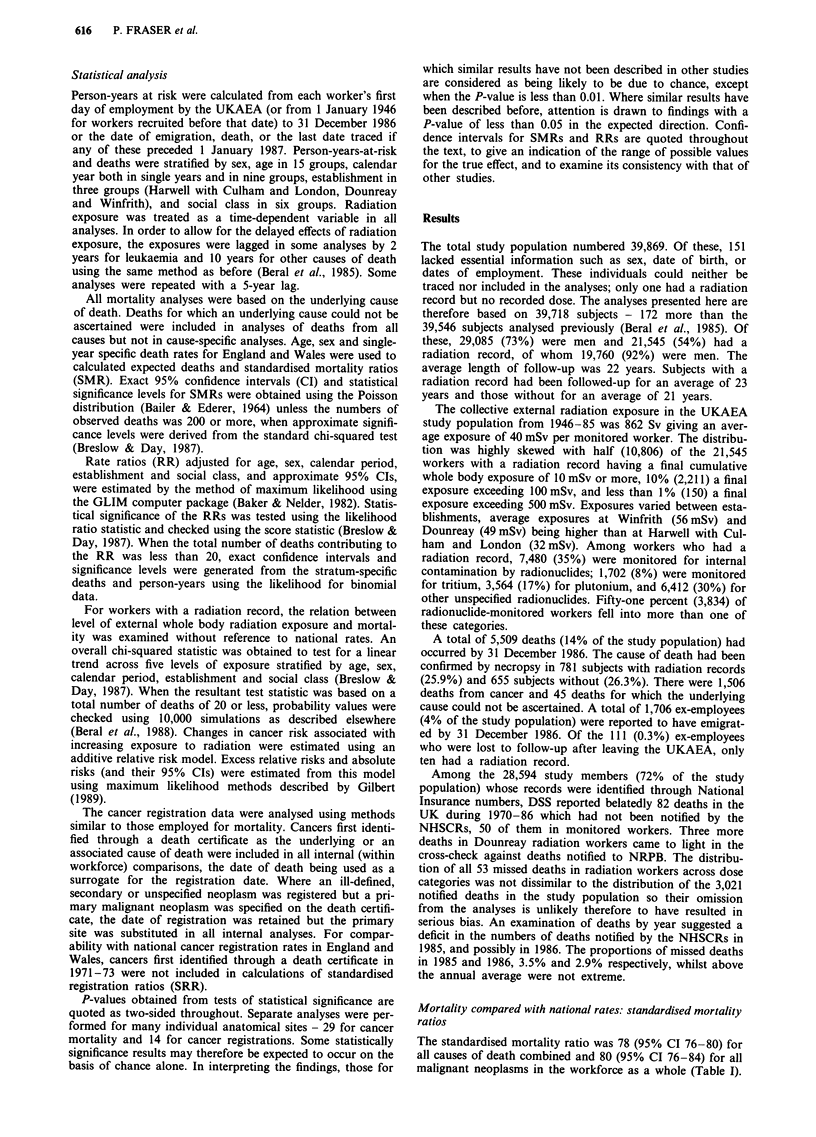

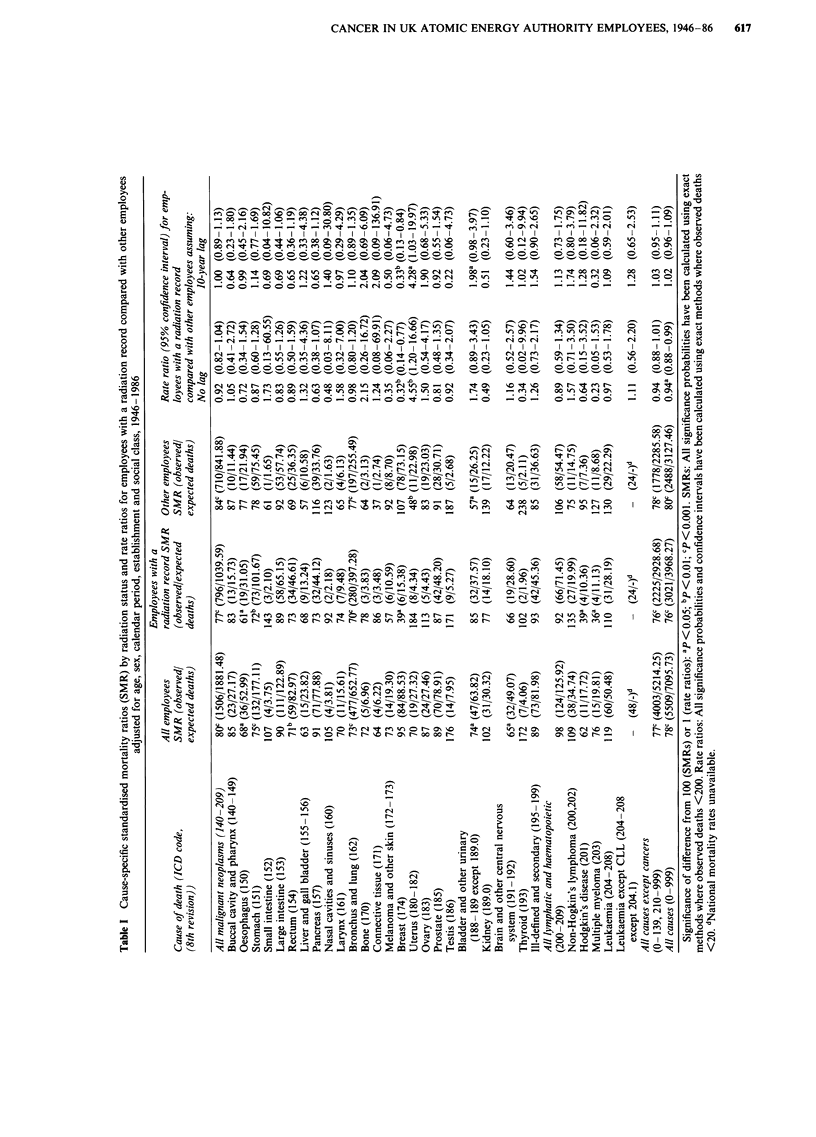

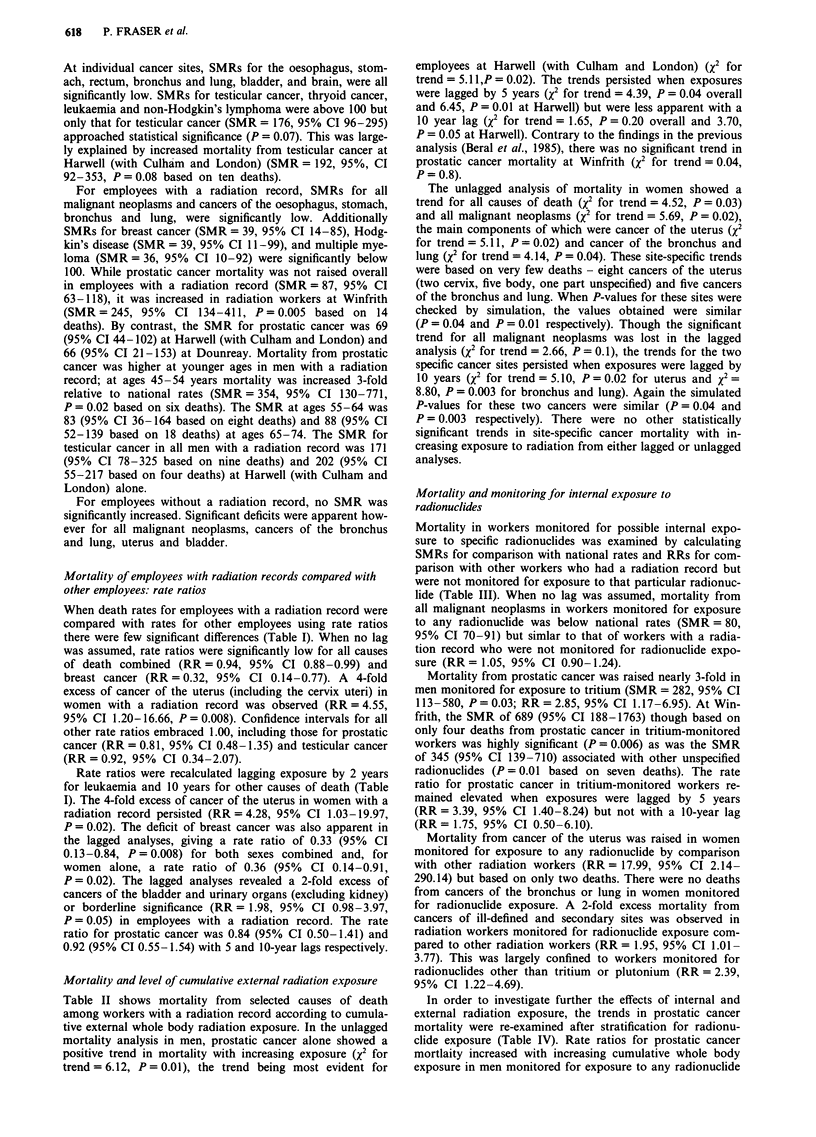

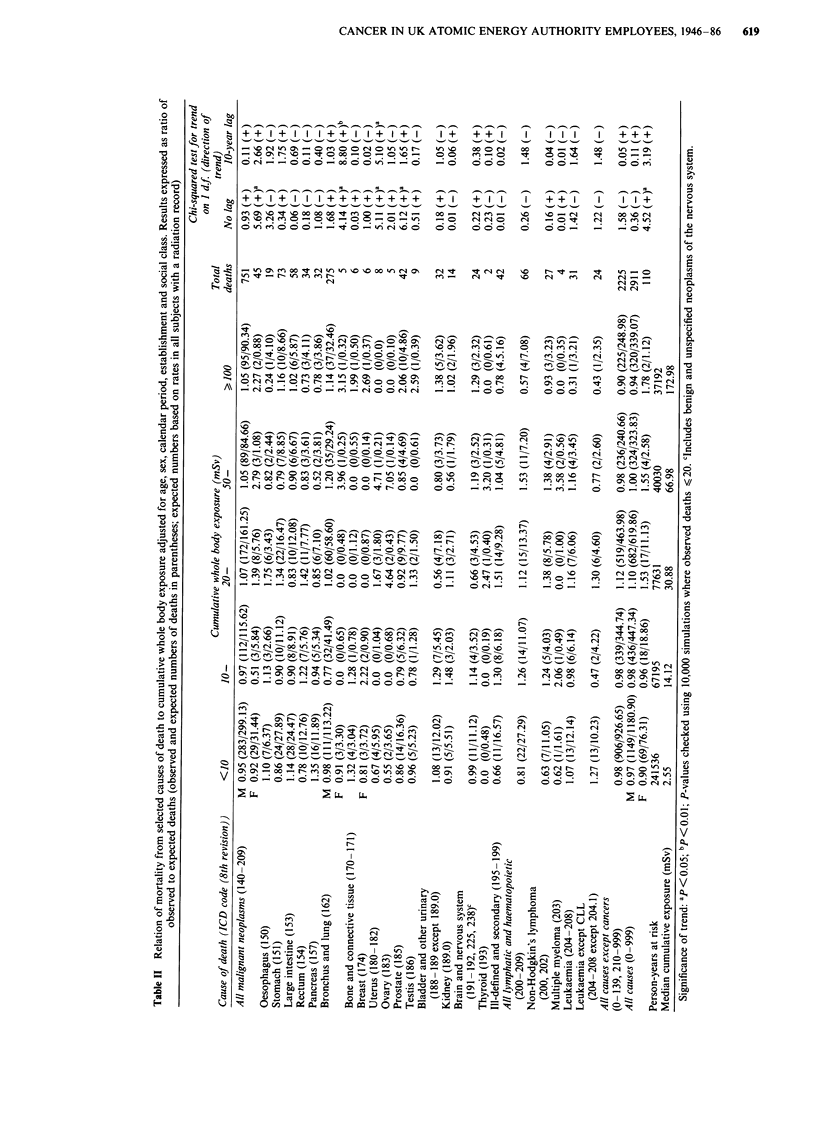

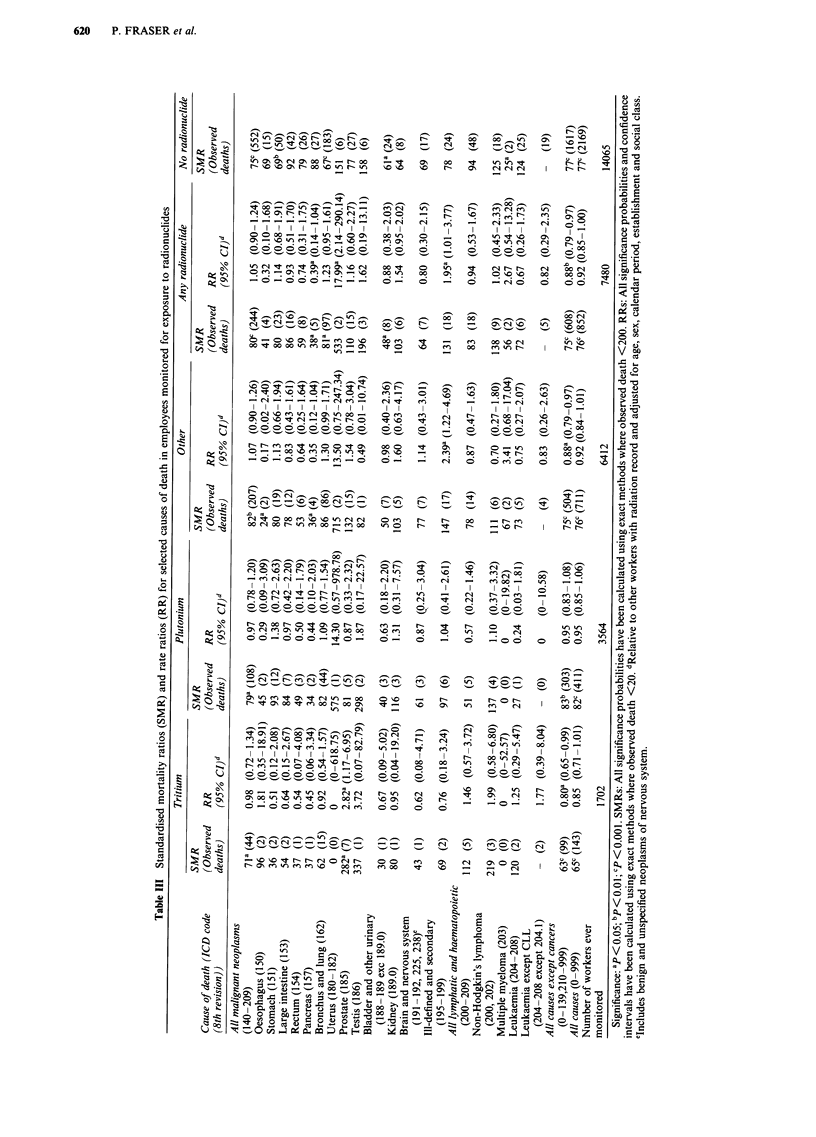

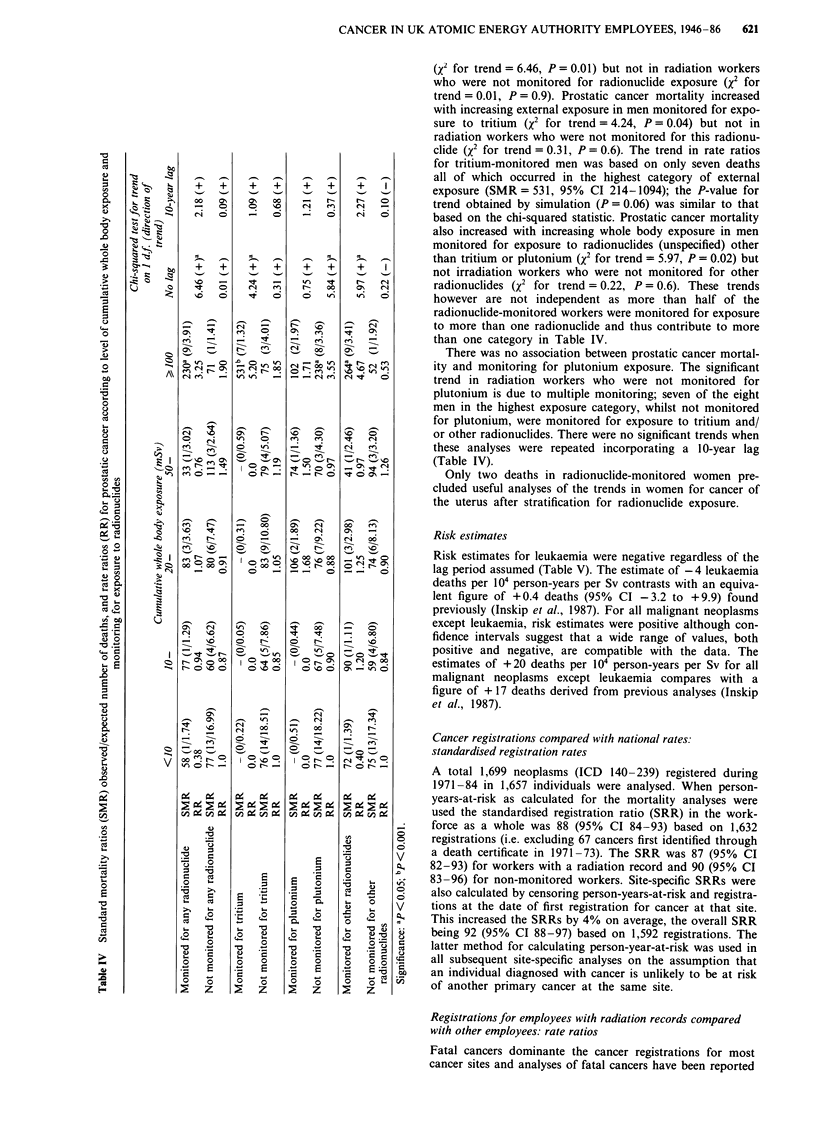

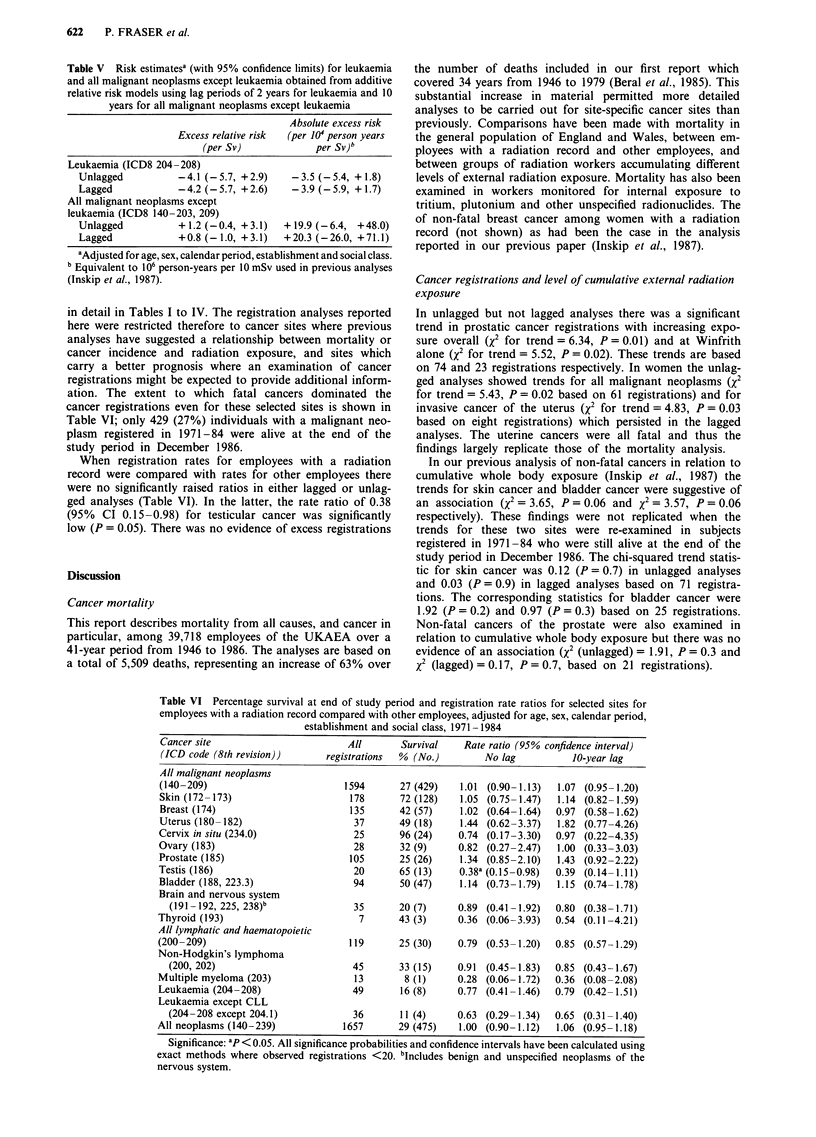

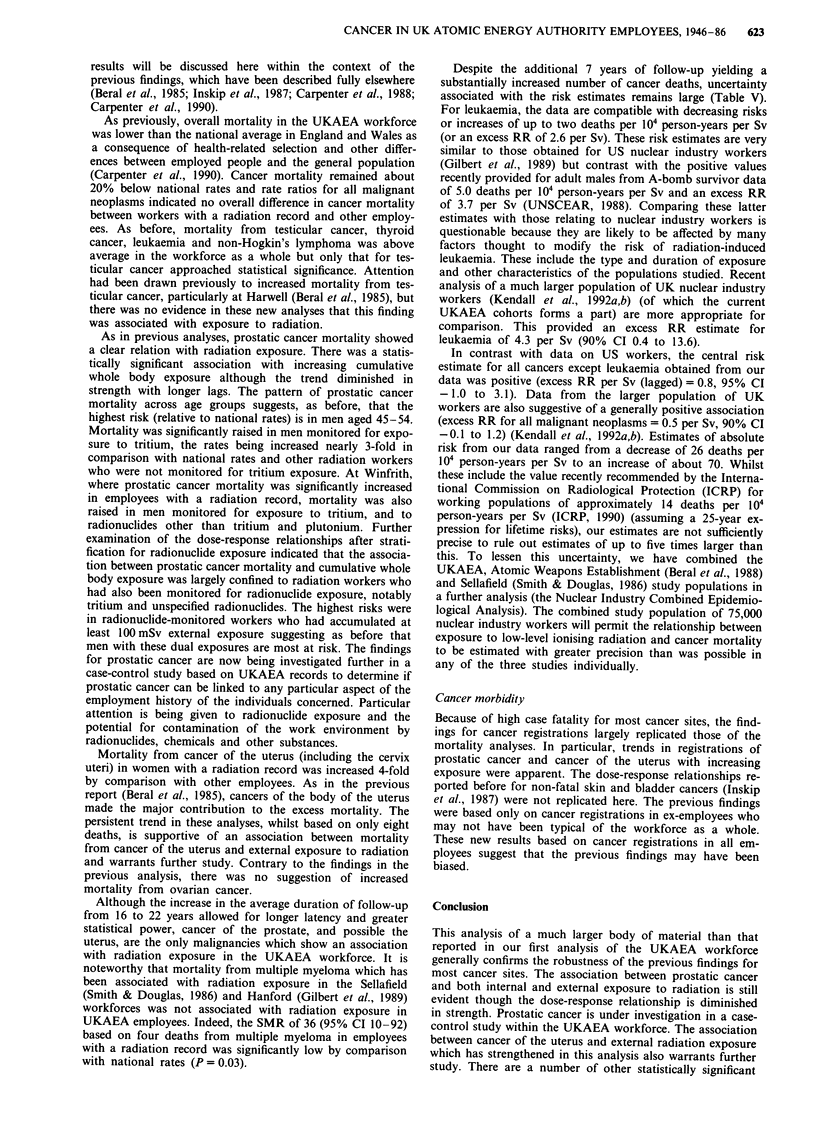

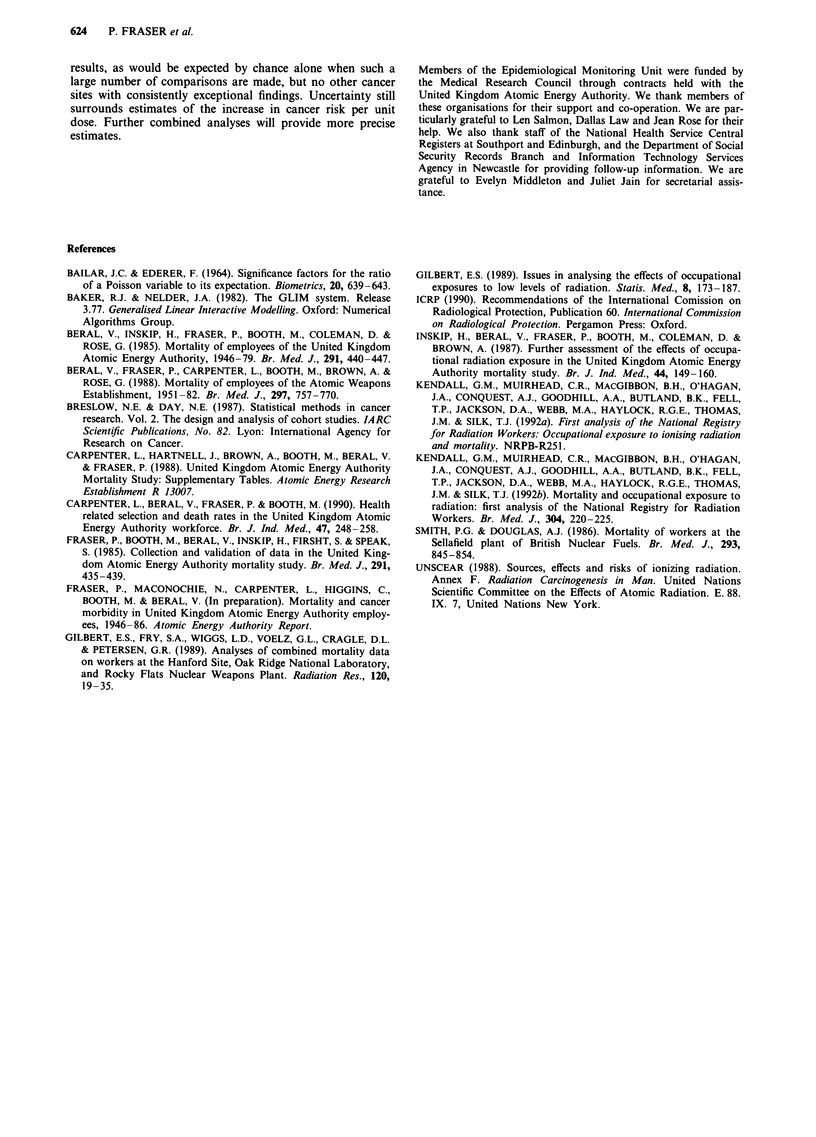

